# General movements and neurodevelopmental outcomes at 2 years of age in infants born very preterm

**DOI:** 10.1111/dmcn.70114

**Published:** 2026-01-06

**Authors:** Ninib Yakoub, Marieken Asprion, Stephanie Brezina, Tilman Reinelt, Giancarlo Natalucci

**Affiliations:** ^1^ Family Larsson‐Rosenquist Foundation Centre for Neurodevelopment, Growth and Nutrition of the Newborn, Department of Neonatology University Hospital Zurich, University of Zurich Zurich Switzerland; ^2^ Newborn Research Zurich, Department of Neonatology University Hospital Zurich, University of Zurich Zurich Switzerland; ^3^ Child Development Center University Children's Hospital Zurich, University of Zurich Zurich Switzerland

## Abstract

**Aim:**

To investigate the relationship between quality of general movements and neurodevelopmental outcomes in 2‐year‐old infants born very preterm (VPT).

**Method:**

This was a retrospective cohort study including infants born before 32 weeks' gestation. General movements video recordings at 3 months corrected age were assessed with the Motor Optimality Score—Revised (MOS‐R) and child cognitive, language, and motor development at 2 years with the Bayley Scales of Infant and Toddler Development, Third Edition.

**Results:**

The study included 316 infants (52.5% male, mean [SD] gestational age 28.7 [2.3] weeks, birthweight z‐score −0.14 [0.85]). The median MOS‐R total score was 23. The mean cognitive composite, language, and motor scores were 102.0 (15.4), 92.1 (16.1), and 95.8 (15.3) respectively. Higher MOS‐R total scores were related to better cognitive (*p* = 0.025) and motor development (*p* = 0.042). However, associations weakened when controlling for socioeconomic status, gestational age, birthweight, sex, and number of severe neonatal morbidities (i.e. severe brain lesion, necrotizing enterocolitis, sepsis, bronchopulmonary dysplasia, and retinopathy of prematurity).

**Interpretation:**

While the quality of general movements at 3 months corrected age is associated with the infant's cognitive and motor development at 2 years corrected age, it might have no incremental predictive power beyond socioeconomic status and the number of severe neonatal morbidities.

## Graphical abstract

This study examines the predictive role of early motor quality in very preterm born infants using the Motor Optimality Score ‐ Revised (MOS‐R). The graphical abstract depicts the link between newborn general movements and cognitive as well as motor outcomes at two years. Findings highlight early motor quality as a measurable indicator for later neurodevelopment.

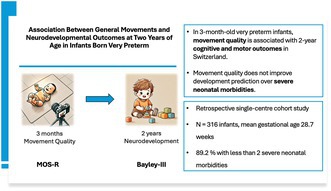

**What this paper adds**

- In 3‐month‐old infants born very preterm, movement quality is associated with 2‐year corrected age cognitive and motor outcomes.
- Movement quality does not improve development prediction beyond socioeconomic status and severe neonatal morbidities.

**Abbreviations:**
MOS‐R: motor optimality score—revised
VPT: very preterm

Worldwide, approximately 10% of newborn infants are born preterm, while 15% of them are born very preterm (VPT; i.e. < 32 weeks of gestation).^1^ Although mortality rates of infants born VPT have decreased over the past decades, there has not been a commensurate decline in morbidity.^2^ Compared to term‐born children, children born VPT have a higher risk of adverse neurological development.^3^ The degree of neurodevelopmental impairment is inversely related to gestational age^4^ impacting cognitive performance, academic achievement, motor skills^3^, cerebral palsy, and behavioural problems.^5^, ^6^, ^7^ Neonatal morbidities, particularly severe brain lesions, necrotizing enterocolitis, sepsis, bronchopulmonary dysplasia, and retinopathy of prematurity, have consistently been related to worse neurodevelopmental outcomes of children born VPT.^8^ Additionally, socioeconomic status is a strong predictor of cognitive development.^9^ Because of the high neural plasticity in early childhood, it is critical to identify and treat children born VPT as early as possible.^10^ The most common early methods for detecting neurodevelopmental impairment are brain imaging and neurodevelopmental assessments.^10^ In addition, the quality of the general movements is an early functional marker of motor development. These endogenously generated movements occur from around 9 weeks of gestation until 20 weeks postterm, when they are gradually replaced by intentional and anti‐gravity movements.^11^ It is assumed that general movements are generated by neural circuits located in the brain stem and modulated by higher level cortical structures.^11^ Brain lesions affecting connectivity and neurodevelopment may cause monotonous and repetitive general movements. Until around 6 weeks corrected age, the general movements have an elliptical form and are characterized by small to moderate amplitude and speed. Between 9 weeks and 20 weeks corrected age, general movements are characterized by small amplitude, moderate speed, and variable acceleration, giving the movements a fidgety character (fidgety movements). General movements can be assessed by Prechtl's General Movements Assessment,^12^ based on Gestalt perception of video recorded movement patterns. The General Movement Assessment was further extended to the Motor Optimality Score—Revised (MOS‐R),^13^ which, in addition to the global assessment of general movements, appraises concurrent motor behaviour, including intentional and anti‐gravity movements. Although the MOS‐R might have incremental validity over the traditional General Movement Assessment when predicting developmental outcomes,^14^, ^15^ the literature is sparse and not conclusive. Studies with the previous version of the Motor Optimality Score^16^ suggest a predictive utility of this score for adverse neurodevelopmental outcomes such as lower intelligence and motor function.^17^ However, the predictive value of the MOS‐R for long‐term neurodevelopment in infants born VPT remains unclear. This study tested whether the quality of general movements, measured by the (1) total and (2) subscale MOS‐R scores, is independently associated with better neurodevelopmental outcomes at 2 years corrected age in children born VPT.

## METHOD

### Study design and participants

This retrospective cohort study included infants born VPT, who underwent a neurodevelopmental examination at a corrected age of 3 months at the University Hospital Zurich between 2014 and 2018. Infants with video recordings of general movements suitable for evaluation according to the standard of the General Movement Assessment^11^ were included. Children with genetic syndromes, severe congenital malformation, inborn metabolic disorders, or prenatal infections that adversely affect life expectancy or neurodevelopment were excluded.

### Procedure

Developmental assessments were conducted as part of the national postdischarge follow‐up programme for children born VPT.^18^ The assessments included recording and evaluating general movements at 3 months corrected age and a neurological and developmental assessment at 2 years corrected age.

The study was approved by the ethics committee of the canton of Zurich, Switzerland (KEK‐ZH‐BASEC‐Nr. 2023–01915). Inclusion in the study was contingent upon parental written informed consent for their child's encrypted neonatal and follow‐up data to be entered into the prospective data register for infants born VPT of the SwissNeoNet.^18^

### Measures

#### Medical data

The following neonatal and demographic data were extracted from the register: gestational age at birth, birthweight and z‐score derived from standardized growth charts,^19^ sex, and multiple pregnancy. In addition, the following severe neonatal morbidities^8^ were extracted: necrotizing enterocolitis of stage II or higher on the Bell classification system, sepsis, moderate to severe bronchopulmonary dysplasia (i.e. need of additional oxygen at 36 weeks postmenstrual age), retinopathy of prematurity stage greater than 2, and severe brain lesions (i.e. intraventricular haemorrhage grade >2 or cystic periventricular leukomalacia). Cranial ultrasound was routinely performed according to the hospital's standard protocol on day 1, 2, 3, and 7 of life and weekly thereafter until discharge. Severe neonatal morbidities were summarized into a composite score where 0 indicates no morbidities and 5 indicates the maximum number of morbidities. Finally, family socioeconomic status was assessed by a validated 12‐point score based on maternal education and paternal occupation, each of which ranged from 1 to 6 points,^9^ a higher score indicating lower socioeconomic status.

#### General movements

Infant general movements and concurrent motor repertoire were assessed using the MOS‐R.^13^ Besides fidgety movements, the motor repertoire contains four additional subscales: movement patterns, age adequate movement repertoire, observed postural patterns, and movement character. All subscales are rated on an ordinal scale. The fidgety movements subscale has the scores 1, 4, or 12 while the other subscales have the scores 1, 2, or 4. The MOS‐R total score, therefore, ranges from 5 to 28, with higher scores indicating age‐appropriate motor behaviour. Based on the MOS‐R classification system,^14^ a total score of 25 to 28 can be categorized as optimal, 20 to 24 as mildly reduced optimality, 9 to 19 as moderately reduced optimality, and 5 to 8 as severely reduced optimality. A referral to early intervention is recommended for a score below 20.

Each video was independently assessed by a rater with an advanced certificate by the General Movements Trust and additionally by one of two extensively trained raters. All raters were blinded to the children's follow‐up outcomes. Disagreements were resolved by consensus with a paediatrician experienced in assessing general movements. Videos were rated if the infant exhibited general movements in an active and calm demeanour for a minimum of 30 seconds. During this sequence, the infant was neither actively engaged, being neurologically assessed, crying nor being fed. If the duration of the sequence adequate for assessment extended 5 minutes, only the first 5 minutes were rated.^20^

#### Child development at 2 years

Following the national standard of the SwissNeoNet,^18^ child cognitive, language, and motor development at 2 years corrected age were assessed by experienced developmental paediatricians using the Bayley Scales of Infant and Toddler Development, Third Edition.^21^ Standardized composite scores for each domain are normalized to a mean of 100 and standard deviation of 15. Higher scores represent better development. Additionally, the presence or absence of cerebral palsy (according to Dan et al.)^22^ and its level of motor deficit according to the Gross Motor Function Classification System^23^ were assessed. Data on therapies received after discharge until 2 years corrected age was read out from hospital records. Physical therapy focused on gross motor skills (posture and mobility), early interventions included additional educational support (such as cognitive stimulation), and occupational therapy emphasized fine motor skills and sensory integration.

### Statistical analysis

Descriptive statistics were presented as mean and standard deviation or median and interquartile range for continuous variables, while frequencies with percentages were presented for categorical variables. Group comparisons were performed using *t*‐tests for continuous variables and χ^2^ tests for categorical variables. Fisher's exact test was applied when expected cell counts were below five. A two‐sided *p*‐value less than 0.05 was considered statistically significant. Little's Missing Completely at Random (MCAR) test^24^ and logistic regression were used to examine whether missingness could be considered at random. As multiple imputation has been advised even if the missing at random assumption has been violated,^25^ multiple imputation has been conducted using the multiple imputation by chained equations package in R (R Foundation for Statistical Computing, Vienna, Austria). Because of the substantial proportion of multiple births (e.g. twins, triplets), multilevel regression models were applied to assess whether the MOS‐R total score or its subscales predicted cognitive, language, or motor outcomes, accounting for the non‐independence of infants from the same families. The multilevel analyses were conducted using linear mixed effects modelling with the lmer package in R. The fixed effects were the MOS‐R scores and control variables, whereby the random effects were the clusters of multiple births. The multilevel models controlled for socioeconomic status, gestational age, birthweight z‐scores, sex, and number of severe neonatal morbidities. Both the number of neonatal morbidities and the socioeconomic status were treated as continuous variables as in previous literature.^8^, ^9^ Continuous predictor variables were mean centred. Furthermore, because of the low frequencies of fidgety movements, observed movement patterns, and movement character in the lower and medium scores of the MOS‐R subscales, the frequencies of the low and middle categories were summarized into an ‘abnormal’ category, as these correspond to clinically relevant atypical movements. The high score ‘normal’ category was used as a reference category. Sensitivity analyses were conducted to assess the robustness of the main effects across different methodological approaches. These approaches included: (1) handling missing data by listwise deletion; (2) including only those covariates that were associated with outcomes at 2 years corrected age; (3) including received therapy at 2 years corrected age in the regression model; (4) treating the MOS‐R as a binary predictor variable with a cut‐off score of less than 20. The inclusion of therapy was based on the assumption that it could influence developmental trajectories, particularly in infants with low general movements quality.

## RESULTS

### Participants

Of 800 infants born VPT and/or hospitalized in the University Hospital Zurich between 2014 and 2018, 677 were eligible and 316 provided codable general movements video recordings (Figure S1).

### Descriptive statistics

Among included infants, 166 (52.5%) were male and 150 (47.5%) were female. The mean (SD) gestational age and birthweight z‐score were 28.7 (2.3) and −0.14 (0.85) respectively. The baseline characteristics were comparable between infants with and without general movements recordings, with the exception of a higher incidence of bronchopulmonary dysplasia and more frequent use of physiotherapy in the former group. At the 2‐year follow‐up, mean developmental scores were 102.0 (SD = 15.4) for cognitive, 92.1 (SD = 16.1) for language, and 95.8 (SD = 15.3) for motor development, 4.7% of the infants were diagnosed with any type of cerebral palsy, and 38.0% received any kind of therapy. Descriptive statistics of the infants included in the analysis compared to eligible infants without video recordings are presented in Table 1.

**Table 1:** Descriptive statistics.

	Study sample (*n* = 316)	No recordings (*n* = 261)	*p*
**Baseline characteristics**			
Gestational age in weeks, mean (SD)	28.7 (2.3)	29.1 (2.1)	
**Sex**			0.521
Male, *n* (%)	166 (52.5)	145 (55.6)	0.521
Female, *n* (%)	150 (47.5)	116 (44.4)	
Multiples, *n* (%)	111 (35.1)	91 (34.9)	> 0.999
Birthweight in grams, mean (SD)	1140 (389)	1210 (362)	0.025
Birthweight z‐score, mean (SD)	−0.144 (0.846)	−0.104 (0.800)	0.563
Small‐for‐gestational age, *n* (%)	37 (11.7)	19 (7.3)	0.105
Antenatal steroids (complete course), *n* (%)	228 (72.2)	194 (74.3)	0.513
**Neonatal morbidities**			
Severe brain lesion, *n* (%)	23 (7.3)	9 (3.4)	0.069
Necrotizing enterocolitis, *n* (%)	2 (0.6)	6 (2.3)	0.149
Sepsis, *n* (%)	31 (9.8)	23 (8.8)	0.790
Retinopathy of prematurity grade >2, *n* (%)	20 (6.3)	10 (3.8)	0.247
Bronchopulmonary dysplasia, *n* (%)	61 (19.3)	26 (10.0)	0.003
Infants with less than two morbidities, *n* (%)	282 (89.2)	245 (93.9)	0.069
Socioeconomic status, median (IQR)	5.0 (3.0–7.0)	5.0 (4.0–6.0)	0.596
**2‐year follow‐up data**			
Composite cognition score, mean (SD)	102.0 (15.4)	102.0 (15.0)	0.892
Composite language score, mean (SD)	92.1 (16.1)	93.1 (17.2)	0.577
Composite motor score, mean (SD)	95.8 (15.3)	97.0 (17.0)	0.528
Cerebral palsy, *n* (%)	15 (4.7%)	10 (3.8%)	0.754
GMFCS level, *n* (%)			
I	7 (2.2)	1 (0.4)	0.061
II	6 (1.9)	4 (1.5)	0.737
III	1 (0.3)	3 (1.1)	0.230
IV	1 (0.3)	0 (0.0)	> 0.999
V	0 (0.0)	2 (0.8)	> 0.999
**Therapies until 2 years**			
Any therapy, *n* (%)	120 (38.0)	67 (25.7)	0.002
Physical therapy, *n* (%)	116 (36.7)	66 (25.3)	0.005
Early intervention, *n* (%)	20 (6.3)	10 (3.8)	0.255
Occupational therapy, *n* (%)	5 (1.6)	3 (1.1)	0.735

*Note*: *t*‐tests were conducted for continuous data and χ^2^ tests for categorical data, whereby all categories were considered. Fisher's exact test was conducted when cells contained less than 5 data points. To compare medians with IQR, Wilcoxon rank‐sum test was conducted. Abbreviations: GMFCS, Gross Motor Function Classification System; IQR, interquartile range; SD, standard deviation.

**Figure 1:**
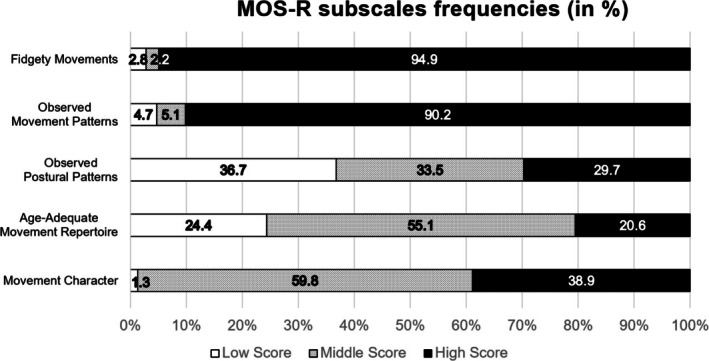
Motor Optimality Score—Revised (MOS‐R) subscales score frequencies (*n* = 316). Low, middle, and high scores correspond to the MOS‐R subscales scores 1, 2, and 4 respectively, except for the subscale fidgety movements, where they correspond to scores of 1, 4, and 12.

The median MOS‐R total score was 23 (range 5–28) with a mean (SD) corrected age at general movements video recording of 13.2 weeks (1.6) and median rateable video duration of 300 seconds (interquartile range: 150.75 seconds). Thirty (9.5%) infants had MOS‐R total scores below the clinical cut‐off (refer to Table S1 for the distribution of MOS‐R classification system categories). Abnormal/absent fidgety movements were observed in 16 (5.1%) infants. Figure 1 shows the frequencies of all MOS‐R subscale scores. While the subscales fidgety movements and observed movement patterns showed high quality of motor behaviour across infants, the subscales observed postural patterns and age‐adequate movement repertoire displayed greater variability across categories. The subscale movement character showed a low frequency of abnormal movement character.

### Association between general movements and neurodevelopmental outcomes at 2 years of age

In univariate analyses, higher values in the MOS‐R total score were significantly associated with better cognitive and motor composite scores (Table 2). These associations weakened after the addition of covariates. A lower socioeconomic status (all β < −0.89, all *p* < 0.021) and a higher number of severe neonatal morbidities were associated with worse infant development across all domains (all β < −4.06, all *p* < 0.004). None of the MOS‐R subscales were associated with developmental outcomes in the multivariate models (Table S2).

**Table 2:** Univariate and multivariate association between MOS‐R and cognitive, language, and motor composite scores of the Bayley Scales of Infant and Toddler Development, Third Edition.

		Cognitive composite			Language composite			Motor composite		
Model	Predictors	b	SE	p	b	SE	p	b	SE	p
**Univariate model**	Intercept	101.73	1.04	< 0.001	92.21	1.10	< 0.001	96.08	1.02	< 0.001
	MOS‐R total score	0.87	0.38	0.025	0.71	0.38	0.060	0.73	0.36	0.042
**Multivariate model**	Intercept	102.79	1.41	< 0.001	93.39	1.47	< 0.001	96.48	1.41	< 0.001
	MOS‐R total score	0.67	0.38	0.079	0.54	0.37	0.151	0.57	0.36	0.114
	Socioeconomic status	−1.53	0.39	< 0.001	−1.92	0.43	< 0.001	−0.89	0.38	0.021
	Sex (m)	−1.60	1.90	0.400	−1.89	1.97	0.338	−0.70	1.92	0.718
	Birthweight z‐score	1.03	1.21	0.395	0.86	1.24	0.490	−0.11	1.20	0.924
	Gestational age (weeks)	−0.08	0.53	0.876	−0.08	0.54	0.878	−0.34	0.52	0.518
	Number of morbidities	−4.75	1.32	0.001	−4.06	1.39	0.004	−5.08	1.37	< 0.001

*Note*: The variables MOS‐R total score, socioeconomic status, gestational age, and number of neonatal morbidities have been centred. Abbreviations: *b*, regression coefficient; MOS‐R, Motor Optimality Score—Revised; SE, standard error.

### Sensitivity and subgroup analyses

Sensitivity analysis using only observed data (listwise deletion) showed positive associations in the multivariate model between the MOS‐R total score and infant cognitive composite score (*b* = 0.54, *t* = 2.02, *p* = 0.046). In all sensitivity analyses, a lower socioeconomic status and a higher number of severe neonatal morbidities consistently indicated lower neurodevelopmental performance. Therapies received after discharge until 2 years corrected age was not a significant predictor or moderator in the multivariate models (Table S3). Predicting outcomes with the MOS‐R cut‐off score of less than 20 instead of the continuous score did not change the pattern of results (Table S4). The only exception was that a MOS‐R score below 20 was associated with worse language scores in univariate models (*b* = −7.41, *t* = −2.02, *p* = 0.044), but not after controlling for confounders.

## DISCUSSION

This study investigated the relationship between the quality of general movements as assessed by the MOS‐R score at 3 months corrected age and neurodevelopmental outcomes at 2 years corrected age as assessed by the Bayley Scales of Infant and Toddler Development, Third Edition in infants born VPT. While higher MOS‐R total scores were associated with better cognitive and motor development, the associations weakened after controlling for covariates. Notably, lower socioeconomic status and the number of severe neonatal morbidities were associated with poorer neurodevelopment, suggesting that parental factors and early perinatal complications may be more predictive of infant development than early motor patterns. The present findings align with previous reports^14^, ^15^, ^26^, ^27^ which identified significant univariate associations between early movement quality and cognitive and motor skills at 2 years of age.

One study^15^ reported a multivariate association between the MOS‐R total score and cognitive and motor development. While our data showed a positive relationship between the MOS‐R and cognitive development, Kwong et al. unexpectedly found a negative association. When comparing the present study with prior research on the relationship between early movement quality and later neurodevelopmental abilities in at‐risk populations, such as infants born preterm, three key methodological differences may account for discrepant results.^15^ These include variations in the timing of general movements assessments, approaches to handling missing data, and strategies for addressing confounders in statistical models. Recent findings^26^ suggest that lower early movement quality is more strongly associated with cognitive and motor impairment at age 2 years in infants born extremely preterm or extremely‐low‐birthweight when assessed at 14 to 15 weeks rather than 12 to 13 weeks corrected age. Thus, the mean corrected age of 13.2 weeks at the time of general movements recording in the present cohort may have reduced the study's power to detect an independent association between the total MOS‐R score and developmental outcomes. Unlike similar studies however, the present one employed a robust method to impute missing data, preserving statistical power and allowing for more precise estimation of relationships between variables.^25^ Finally, although some studies consider predictors of cognitive and motor development such as sociodemographic and perinatal characteristics, severe neonatal morbidities—recognized risk factors for poor neurodevelopmental outcomes^8^—have been rarely included in analyses before.

The absence of an independent association between early movement quality and later development in the present study may be attributed to strong neurodevelopmental predictors such as socioeconomic status and the number of severe neonatal morbidities. While evaluating the quality of infants' movements requires specialized training and considerable resources for large‐scale implementation, accounting for severe morbidities in infants born preterm is far less resource intensive. It remains unclear, however, how supportive therapies received may have weakened the relationship between MOS‐R and neurodevelopmental outcomes. In the present study, all infants received physiotherapy during their hospitalization in the neonatal intensive care unit and their parents received a basic handling instruction as standard care for this patient group. After discharge, more than a third of the infants received some kind of developmental support between hospital discharge and the follow‐up visit. Based on existing evidence,^28^ it is possible that early exposure to developmental care may have positively influenced the developmental trajectories of infants. Although the sensitivity analysis did not reveal any significant association or interaction effect between received therapies and outcomes, the observational and retrospective nature of the study may limit the ability to detect such effects.

Some limitations reduce the generalizability of the results. First, the retrospective study design with a convenience sample might question the representativeness of the results for the general population of children born VPT. Although the baseline characteristics of the participants were similar to those of eligible but non‐included infants, the representativeness of the cohort is limited by its low‐risk profile and relatively favourable 2‐year outcomes. As a result, the underrepresentation of infants with poorer developmental outcomes in the 2‐year follow‐up may have reduced the likelihood of detecting a true association. Furthermore, the low prevalence of abnormal scores in MOS‐R subscales such as fidgety movements limited the power needed to perform a robust analysis. In addition, because of their low prevalences, neonatal morbidities had to be aggregated into a single composite score.^8^

Despite these limitations, this study has strengths including a relatively large sample size. In addition, baseline data and follow‐up data at age 2 years were extracted from a prospective registry with high‐quality criteria and the follow‐up examinations at both 3 months and 2 years were conducted in accordance with a clearly defined standard. Future research should investigate potential moderators and mediators that may affect the association between quality of general movements in infancy and neurodevelopment at 2 years of age. For instance, one study reported that therapeutic hypothermia may alter the prognostic value of general movements for cerebral palsy.^29^ Special attention should be given to the quality and quantity of care both in neonatal intensive care units and after hospital discharge, as they may impact the prevalence of neurodevelopmental impairments, potentially affecting statistical power in studies by altering the strength of observed associations. In addition, longitudinal studies are needed to investigate possible associations of general movement quality and child development beyond toddlerhood. A recent study suggested that general movements may predict child development at school age.^17^, ^30^ However, given that neurodevelopmental trajectories in early childhood are not stable, and that the MOS‐R is a relatively new assessment tool, further studies are needed to establish its predictive validity for later developmental outcomes. Finally, despite not confirming our hypotheses, the MOS‐R may still hold relevance for at‐risk infants by offering an additional perspective that supports clinical judgement regarding a child's developmental status and therapeutic needs. As the MOS‐R is based on video recordings, it has been suggested as a valuable screening instrument for identifying neurodevelopmental risks in remote settings where in‐person evaluations are challenging or when accurate screening for major morbidities is limited.^17^, ^26^

## CONCLUSION

The quality of general movements may be associated with neurodevelopmental outcomes at 2 years corrected age. As such, assessing general movements quality continues to be a valuable tool for screening infants with early functional motor problems. However, it may not provide additional predictive value for neurodevelopmental outcomes beyond that offered by socioeconomic status and the burden of severe neonatal morbidities. To better understand how early motor patterns relate to later development, more research is needed on how low socioeconomic status, neonatal morbidities, and supportive interventions influence this association. This would help to clarify when the MOS‐R is most effective for identifying infants at higher risk for neurodevelopmental impairments.

## Supporting information


**Figure S1:** Study flowchart

**Table S1:** MOS‐R total score categorization according to the MOS‐R classification system

**Table S2:** Multivariate association between MOS‐R subscales and cognitive, language, and motor composite scores of the Bayley‐III

**Table S3:** Multivariate association between MOS‐R and cognitive, language, and motor composite scores of the Bayley‐III, including therapy as predictor or interaction effect

**Table S4:** Multivariate association between MOS‐R and cognitive, language, and motor composite scores of the Bayley‐III, with MOS‐R total score as binary variable

## Data Availability

The data that support the findings of this study are available on request from the corresponding author. The data are not publicly available due to privacy or ethical restrictions.
