# Supplementary figures and images for: General movements and neurodevelopmental outcomes at 2 years of age in infants born very preterm
Source: Dev Med Child Neurol. 2026 Jan 6;68(8):1097–104. doi: 10.1111/dmcn.70114 (PMC13340619; doi:10.1111/dmcn.70114)

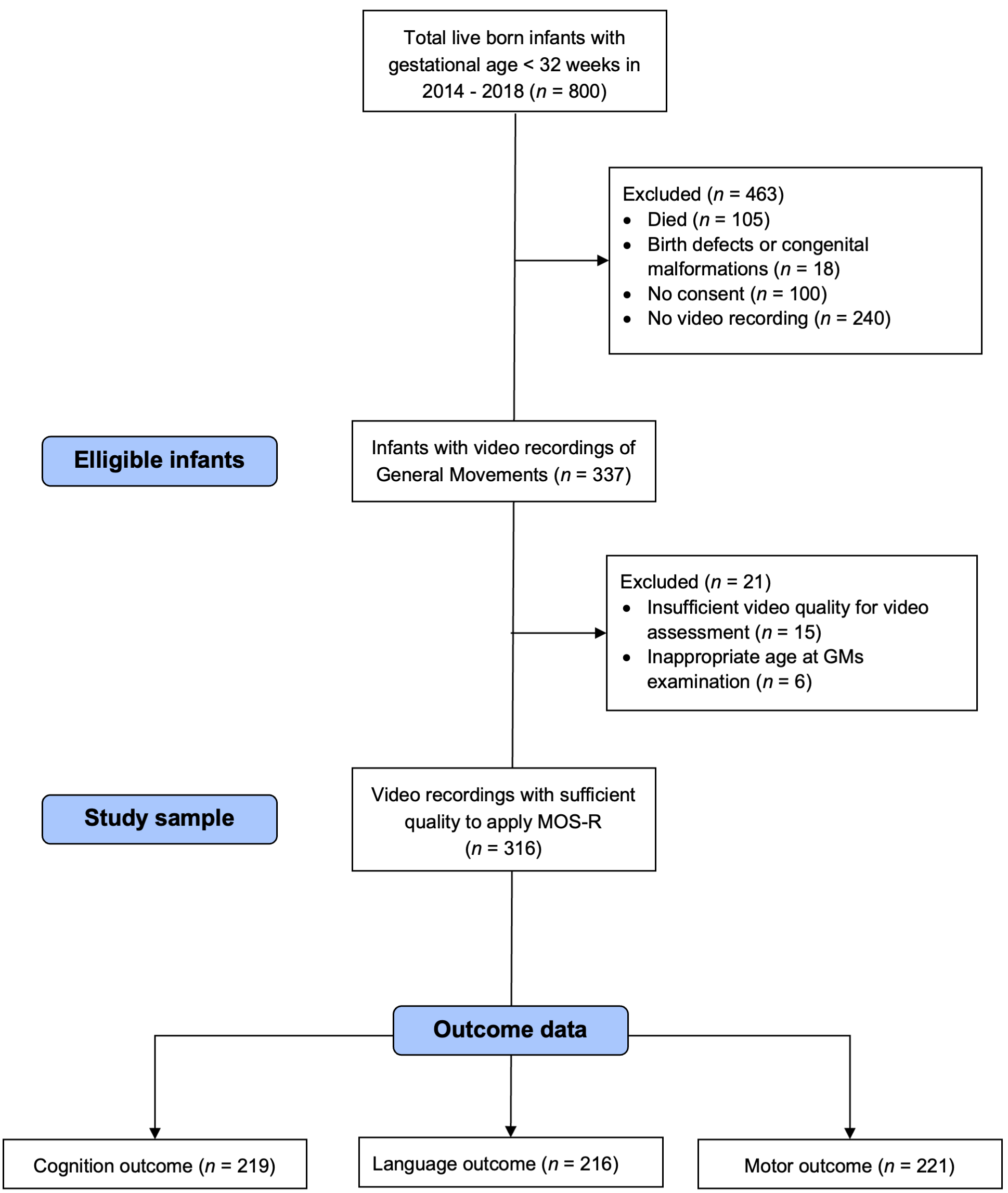

Supplement: Supplementary file 1 — Figure S1: Study flowchart [file DMCN-68-1097-s003.png]
