# Supplementary material for: General movements and neurodevelopmental outcomes at 2 years of age in infants born very preterm
Source: Dev Med Child Neurol. 2026 Jan 6;68(8):1097–104. doi: 10.1111/dmcn.70114 (PMC13340619; doi:10.1111/dmcn.70114)
Supplement: Supplementary file 2 — Table S1: MOS‐R total score categorization according to the MOS‐R classification system [file DMCN-68-1097-s002.docx]

**Table S1: MOS-R total score categorization according to the MOS-R classification system**

| MOS-R score | Optimality category | n (%) |
| --- | --- | --- |
| 25-28 | Optimal | 63 (19.9) |
| 20-24 | Mildly reduced | 223 (70.6) |
| 9-19 | Moderately reduced | 26 (8.2) |
| 5-8 | Severely reduced optimality | 4 (1.3) |

Referral to early intervention recommended (if not already engaged) with an MOS-R score below 20.
