# Supplementary material for: General movements and neurodevelopmental outcomes at 2 years of age in infants born very preterm
Source: Dev Med Child Neurol. 2026 Jan 6;68(8):1097–104. doi: 10.1111/dmcn.70114 (PMC13340619; doi:10.1111/dmcn.70114)
Supplement: Supplementary file 3 — Table S2: Multivariate association between MOS‐R subscales and cognitive, language, and motor composite scores of the Bayley‐III [file DMCN-68-1097-s001.docx]

**Table S2: Multivariate association between MOS-R sub-scales and cognitive, language, and motor composite scores of the Bayley Scales of Infant and Toddler Development, 3^rd^ edition**

|  | Cognitive Composite | | | Language Composite | | | Motor Composite | | |
| --- | --- | --- | --- | --- | --- | --- | --- | --- | --- |
| Predictors​ | **b** | ***SE*​** | ***p*-value​** | ***b*** | ***SE*​** | ***p*-value​** | ***b*** | ***SE*​** | ***p*-value​** |
| Intercept​ | 106.97 | 3.38 | <.001 | 96.10 | 3.55 | <.001 | 98.20 | 3.34 | <.001 |
| Abnormal MOS-R FM | -6.74 | 4.48 | .134 | -6.29 | 4.53 | .167 | -4.36 | 4.64 | .349 |
| Abnormal MOS-R MP | -0.69 | 3.55 | .846 | -1.49 | 3.53 | .673 | -3.51 | 3.54 | .323 |
| MOS-R PP score 1​ | -1.26 | 2.54 | .620 | -1.25 | 2.62 | .634 | -2.88 | 2.46 | .243 |
| MOS-R PP score 2​ | -1.97 | 2.39 | .412 | -0.64 | 2.55 | .803 | -1.11 | 2.37 | .639 |
| MOS-R AAR score 1​ | -2.09 | 3.06 | .496 | -1.54 | 3.13 | .623 | -0.42 | 3.03 | .891 |
| MOS-R AAR score 2​ | -0.99 | 2.49 | .691 | -0.96 | 2.60 | .712 | 0.87 | 2.53 | .731 |
| Abnormal MOS-R MC | -2.54 | 2.26 | .262 | -1.11 | 2.43 | .649 | -0.40 | 2.19 | .856 |
| Socio-economic status | -1.56 | 0.40 | <.001 | -1.93 | 0.44 | <.001 | -0.88 | 0.39 | .025 |
| Sex (m)​ | -1.66 | 1.91 | .385 | -1.88 | 1.97 | .340 | -0.47 | 1.93 | .810 |
| Birth weight z score​ | 0.99 | 1.24 | .425 | 0.82 | 1.27 | .519 | -0.16 | 1.22 | .897 |
| Gestational age (w)​ | -0.10 | 0.54 | .850 | -0.10 | 0.55 | .855 | -0.28 | 0.53 | .595 |
| Number of morbidities | -4.92 | 1.34 | <.001 | -4.06 | 1.40 | .004 | -4.89 | 1.38 | <.001 |

*b*, regression coefficient; *SE*, standard error; MOS-R, Motor Optimality Score – Revised; FM, Fidgety Movements; MP, Observed Movement Patterns; PP, Observed Postural Patterns, AAR, Age-Adequate Movement Repertoire; MC, Movement Character. The variables socio-economic status, gestational age and number of neonatal morbidities have been centred. MOS-R sub-scale scores of 1 and 2 correspond to the low and middle rating category.
