# Supplementary material for: General movements and neurodevelopmental outcomes at 2 years of age in infants born very preterm
Source: Dev Med Child Neurol. 2026 Jan 6;68(8):1097–104. doi: 10.1111/dmcn.70114 (PMC13340619; doi:10.1111/dmcn.70114)
Supplement: Supplementary file 4 — Table S3: Multivariate association between MOS‐R and cognitive, language, and motor composite scores of the Bayley‐III, including therapy as predictor or interaction effect [file DMCN-68-1097-s005.docx]

**Table S3: Multivariate association between MOS-R and cognitive, language, and motor composite scores of the Bayley Scales of Infant and Toddler Development, 3^rd^ edition, including therapy as predictor or interaction effect**

|  |  | Cognitive Composite | | | Language Composite | | | | Motor Composite | | | |
| --- | --- | --- | --- | --- | --- | --- | --- | --- | --- | --- | --- | --- |
| Model | **Predictors​** | ***b*** | ***SE*​** | ***p*-value​** | | ***b*** | ***SE*​** | ***p*-value​** | | ***b*** | ***SE*​** | ***p*-value​** |
| Model with therapy as predictor | Intercept​ | 103.95 | 1.70 | <.001 | | 94.55 | 1.77 | <.001 | | 98.44 | 1.69 | <.001 |
|  | MOS-R total score​ | 0.65 | 0.38 | .092 | | 0.51 | 0.37 | .172 | | 0.53 | 0.36 | .143 |
|  | Therapy at two years | -2.85 | 2.03 | .161 | | -2.83 | 2.10 | .180 | | -4.76 | 2.03 | .020 |
|  | Socio-economic status | -1.56 | 0.39 | <.001 | | -1.95 | 0.43 | <.001 | | -0.93 | 0.38 | .015 |
|  | Sex (m)​ | -1.72 | 1.90 | .365 | | -2.01 | 1.96 | .307 | | -0.90 | 1.91 | .636 |
|  | Birth weight z score​ | 0.86 | 1.22 | .480 | | 0.69 | 1.24 | .581 | | -0.41 | 1.20 | .734 |
|  | Gestational age (w)​ | -0.15 | 0.53 | .775 | | -0.15 | 0.54 | .778 | | -0.45 | 0.52 | .384 |
|  | Number of morbidities | -4.27 | 1.36 | .002 | | -3.58 | 1.43 | .013 | | -4.28 | 1.41 | .003 |
| Model with therapy as interaction effect | Intercept​ | 103.97 | 1.70 | <.001 | | 94.65 | 1.78 | <.001 | | 98.49 | 1.69 | <.001 |
|  | MOS-R total score​ | 0.60 | 0.60 | .320 | | 0.28 | 0.58 | .635 | | 0.42 | 0.56 | .458 |
|  | Therapy at two years | -2.85 | 2.03 | .161 | | -2.84 | 2.10 | .179 | | -4.76 | 2.03 | .020 |
|  | Socio-economic status | -1.56 | 0.39 | .000 | | -1.94 | 0.43 | <.001 | | -0.93 | 0.38 | .015 |
|  | Sex (m)​ | -1.72 | 1.89 | .365 | | -1.99 | 1.96 | .310 | | -0.90 | 1.91 | .638 |
|  | Birth weight z score​ | 0.86 | 1.22 | .480 | | 0.69 | 1.24 | .579 | | -0.41 | 1.20 | .735 |
|  | Gestational age (w)​ | -0.14 | 0.53 | .794 | | -0.09 | 0.55 | .873 | | -0.42 | 0.52 | .416 |
|  | Number of morbidities | -4.22 | 1.36 | .002 | | -3.34 | 1.44 | .021 | | -4.18 | 1.40 | .003 |
|  | MOS-R:Therapy | 0.09 | 0.63 | .886 | | 0.45 | 0.62 | .470 | | 0.20 | 0.62 | .742 |

*b*, regression coefficient; *SE*, standard error; MOS-R, Motor Optimality Score – Revised. The variables MOS-R total score, socio-economic status, gestational age and number of neonatal morbidities have been centred.
