# Supplementary material for: General movements and neurodevelopmental outcomes at 2 years of age in infants born very preterm
Source: Dev Med Child Neurol. 2026 Jan 6;68(8):1097–104. doi: 10.1111/dmcn.70114 (PMC13340619; doi:10.1111/dmcn.70114)
Supplement: Supplementary file 5 — Table S4: Multivariate association between MOS‐R and cognitive, language, and motor composite scores of the Bayley‐III, with MOS‐R total score as binary variable [file DMCN-68-1097-s004.docx]

**Table S4: Multivariate association between MOS-R and cognitive, language, and motor composite scores of the Bayley Scales of Infant and Toddler Development, 3^rd^ edition, with MOS-R total score as binary variable (cut-off <20)**

|  |  | **Cognitive Composite** | | | **Language Composite** | | | | **Motor Composite** | | | | |
| --- | --- | --- | --- | --- | --- | --- | --- | --- | --- | --- | --- | --- | --- |
| **Model** | **Predictors​** | ***b*** | ***SE*​** | ***p*​** | | ***b*** | ***SE*​** | ***p*​** | | ***b*** | ***SE*​** | ***p*** |  |
| **Univariate Model** | Intercept​ | 102.51 | 1.12 | <.001 | | 92.90 | 1.20 | <.001 | | 96.94 | 1.10 | <.001 |  |
|  | MOS-R binary (Low) | -8.29 | 3.80 | .031 | | -7.41 | 3.66 | .044 | | -9.17 | 3.54 | .01 |  |
| **Multivariate Model** | Intercept​ | 103.46 | 1.45 | <.001 | | 94.00 | 1.52 | <.001 | | 97.21 | 1.46 | <.001 |  |
|  | MOS-R binary (Low) | -5.86 | 3.77 | .122 | | -5.53 | 3.59 | .125 | | -6.85 | 3.59 | .058 |  |
|  | Socio-economic status | -1.57 | 0.40 | .000 | | -1.95 | 0.43 | <.001 | | -0.93 | 0.39 | .016 |  |
|  | Sex (m)​ | -1.83 | 1.89 | .335 | | -2.05 | 1.95 | .295 | | -0.85 | 1.91 | .659 |  |
|  | Birth weight z score​ | 1.13 | 1.22 | .353 | | 0.94 | 1.25 | .453 | | -0.04 | 1.20 | .972 |  |
|  | Gestational age (w)​ | -0.11 | 0.53 | .834 | | -0.11 | 0.54 | .842 | | -0.37 | 0.52 | .483 |  |
|  | Number of morbidities | -4.73 | 1.34 | .001 | | -3.97 | 1.40 | .005 | | -4.92 | 1.39 | <.001 |  |

*b*, regression coefficient; *SE*, standard error; MOS-R, Motor Optimality Score – Revised. The variables socio-economic status, gestational age and number of neonatal morbidities have been centred.
